# Simulation of the Recharging Method of Implantable Biosensors Based on a Wearable Incoherent Light Source

**DOI:** 10.3390/s141120687

**Published:** 2014-11-03

**Authors:** Yong Song, Qun Hao, Xianyue Kong, Lanxin Hu, Jie Cao, Tianxin Gao

**Affiliations:** 1 School of Opto-Electronics, Beijing Institute of Technology, Beijing 100081, China; E-Mails: 2120130607@bit.edu.cn (X.K.); 1120100944@bit.edu.cn (L.H.); 3120110258@bit.edu.cn (J.C.); 2 School of Life Science, Beijing Institute of Technology, Beijing 100081, China; E-Mail: gtx@bit.edu.cn

**Keywords:** implantable biosensor, incoherent light source, recharging method, Monte Carlo method

## Abstract

Recharging implantable electronics from the outside of the human body is very important for applications such as implantable biosensors and other implantable electronics. In this paper, a recharging method for implantable biosensors based on a wearable incoherent light source has been proposed and simulated. Firstly, we develop a model of the incoherent light source and a multi-layer model of skin tissue. Secondly, the recharging processes of the proposed method have been simulated and tested experimentally, whereby some important conclusions have been reached. Our results indicate that the proposed method will offer a convenient, safe and low-cost recharging method for implantable biosensors, which should promote the application of implantable electronics.

## 1. Introduction

Recently, more and more implantable biosensors are being implanted into the human body in the medical field. As a result, high efficiency medical monitoring can be implemented directly within the human body [1,2]. Implantable biosensors are strongly dependent on electric power for their continuous operation [3], but current battery technologies fail to completely meet the requirements of implantable biosensors. As a result, patients have to replace new implant batteries by operation every 2–5 years. Therefore, methods for recharging implantable biosensors *in vitro* have become an important issue that has attracted more and more research interest [3–7].

Any recharging method applied to electronics implanted into the human body must meet the requirements of safety, high energy efficiency and convenience [8]. However, it is difficult for the existing previous recharging methods for implantable biosensors to meet these requirements. For instance, in the radio frequency electromagnetic induction method [9,10], which is considered as an important recharging method for implantable biosensors, the heat produced in the electromagnetic induction process interferes with the physiological activity of the human body, while both the operation of biosensors and the electromagnetic induction process itself can suffer interference from the electromagnetic environment of nearby electromagnetic emitting devices [11]. In the recharging methods based on nanowires [4,6], piezoelectric devices [12] and thermo electric generators, only very low power can be achieved [8,12–14]. Meanwhile, the mechanical deformation caused by these methods may also be harmful to the human body [9]. Compared with the above methods, the optical recharging method has the advantages of high energy efficiency as well as low electromagnetic interference [8,15], making it a promising method for recharging implantable biosensors. However, in the current optical recharging method, a laser source, which is a coherent light source, is generally used to achieve the required power density in tissue [8,15], which results in a light source of comparatively big size and high cost. Moreover, the laser source also has the potential to be harmful to skin tissue [16]. The above factors limit the application of the optical method.

On the other hand, a Light Emitting Diode (LED) light source, which is an incoherent light source, has the characteristics of small size, low cost and high safety [17]. If LED light sources could be used for recharging biosensors, it would help decrease the size and cost of the light source, as well as improve the safety of the optical recharging method.

In order to recharge implantable biosensors using a wearable LED light source, the structure and parameters of light source used, which influence the recharging efficiency as well as the safety of the human body to some extent, should be analyzed. Meanwhile, the distribution of the optical power of the incoherent light within skin tissue also should be obtained. Unfortunately, very few works have been reported about the use of LED light sources for recharging biosensors. Moreover, as for the simulation of light propagation within skin tissue, previous works mainly concentrated on the simulation of light with a single wavelength, rather than that of the multi-wavelength light. As a result, the distribution of the optical power of the incoherent light remains undetermined.

In this paper, we propose a method for recharging implantable biosensors from the outside of the human body using a wearable LED light source. Firstly, we develop a model of the LED light source and a multi-layer model of skin tissue. Secondly, the recharging processes of the proposed method have been simulated and verified experimentally, while some important conclusions have been reached, which indicate that the proposed method could provide a convenient, safe and low-cost recharging method for implantable biosensors.

## 2. Method

**Figure 1.**
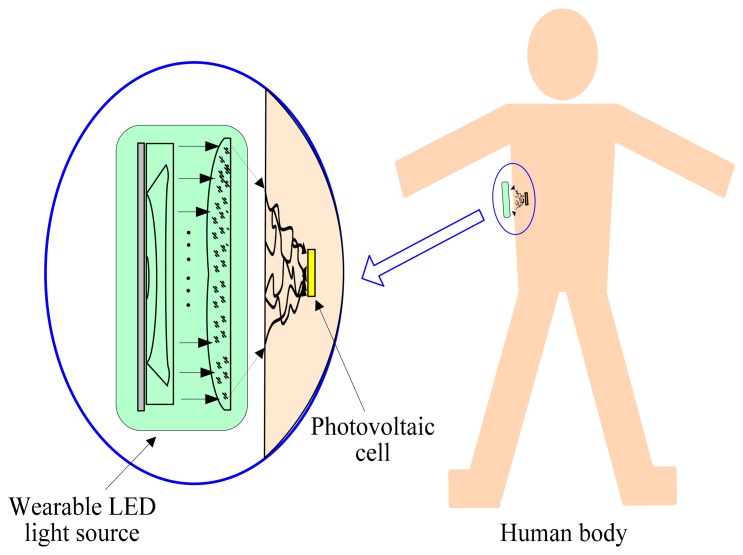
The recharging method for implantable biosensors using a LED light source.

As shown in Figure 1, in the proposed recharging method for implantable biosensors, we use a wearable LED light source to avoid the adverse effects of the previous recharging methods based on laser sources. Firstly, the wearable LED light source emits multi-wavelength light, and the light is focused by a planoconvex lens. Then, the focused light arrives at the surface of skin, and transmits within skin tissue. Finally, some of the light arrives at the photovoltaic cell, and the corresponding energy can be produced by the photoelectric conversion of the photovoltaic cell. Therefore, recharging for implantable biosensors can be implemented using the a wearable LED light source.

## 3. Modeling

### 3.1. Incoherent Light Source Model

#### 3.1.1. Spectrum

In the spectrum selection of the LED light source used in the proposed method, we mainly considered two issues: (1) due to the fact that the penetration depth of light in skin tissue is related to wavelength, the spectrum selection should help to achieve deeper penetration depth in skin tissue; (2) in order to achieve higher photoelectric conversion efficiency, the selected spectrum should match the spectrum of the photovoltaic cell. Generally, near-infrared light with a central wavelength of 1100 nm or 850 nm has a comparatively deeper transmission depth in skin tissue [18]. Moreover, as for most photovoltaic cells, the responsivity to 850 nm light is higher than that to 1100 nm light. Therefore, 850 nm was set as the central wavelength of the LED light source in our investigation.

#### 3.1.2. Structure

**Figure 2.**
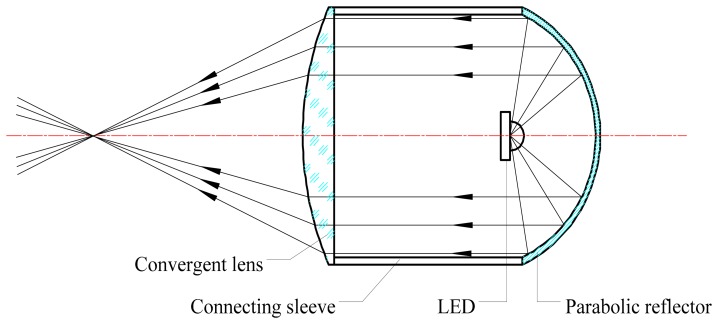
The structure of the LED light source.

As shown in Figure 2, the LED light source includes a LED, a parabolic reflector, a connecting sleeve and a convergent lens, of which the LED can be treated as a point light source located at the object focal point, while the scattering angle *θ* is 60°. Firstly, the emitted light from the LED is converted into parallel light by the parabolic reflector. Subsequently, the parallel light is converged by the planoconvex lens.

#### 3.1.3. Parameters

**Figure 3.**
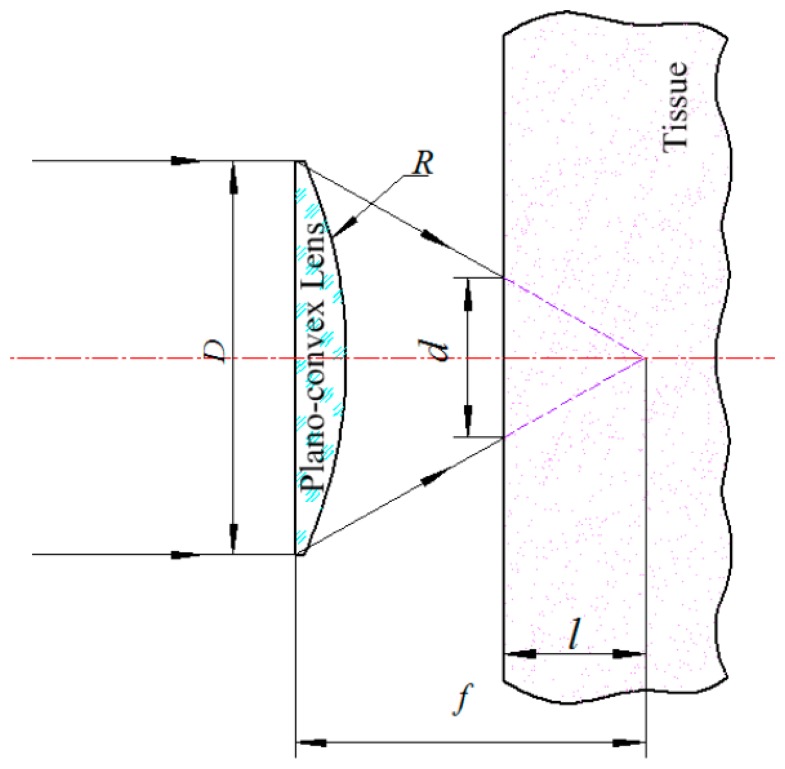
The parameters of the LED light source used for recharging implantable biosensors.

Figure 3 shows the parameters of the LED light source used for recharging the implantable biosensors. The spot area (*S_r_*) at skin surface can be expressed by Equation (1), where *d* is the diameter of the spot at the skin surface, *l* is the distance between the skin surface and the focus of the planoconvex lens, while *D*, *n*, *R* are the aperture, refraction index and curvature radius of the planoconvex lens, respectively. Then the power density (*I*), which represents the incident optical power corresponding to the unit area of skin surface, can be represented as Equation (2), where *P* is the optical power corresponding to *S_r_*:

(1) $$S_{r}=\pi {\left(\frac{d}{2}\right)}^{2}=\frac{1}{4}\pi {\left[\frac{Dl(n-1)}{R}\right]}^{2}$$

(2) $$I=\frac{P}{S_{r}}=\frac{4P}{\pi d^{2}}=\frac{4PR^{2}}{\pi l^{2}{\left(n-1\right)}^{2}D^{2}}$$

### 3.2. Skin Tissue Model

**Figure 4.**
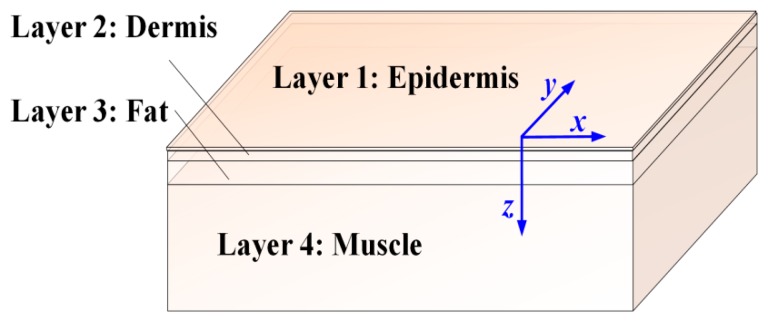
The human tissue model with the layers of epidermis, dermis, fat and muscle.

In our investigation, human tissue was modeled geometrically by four infinitely wide layers, which includes the epidermis, dermis, fat and muscle, as shown in Figure 4. The thicknesses of the epidermis, dermis and fat layers are 0.0065 mm, 0.125 mm and 1.2 mm, respectively [19], while the thickness of muscle is assumed to be infinite because generally light cannot penetrate this layer. Moreover, a Cartesian coordinate system shown in Figure 4 is used for tracing photon movements, of which the *xy* plane is parallel to the tissue surfaces. The direction of the *z*-axis is perpendicular to the tissue surfaces and parallel to the original incident direction of photons.

The geometry and optical properties of the skin tissue model are described with five parameters, which include the thickness (*d*), the absorption coefficient (*μ_a_*), the scattering coefficient (*μ_s_*), the anisotropy factor (*g*) and the relative refractive index (*n_rel_*). The parameters used in our simulations are listed in Table 1 [20–24], in which *μ_a_*, *μ_s_* and *g* are corresponding to the wavelengths of 800 nm, 825 nm, 850 nm, 875 nm and 900 nm. As shown in Table 2, the *μ_a_*, *μ_s_* and *g* of the three layers (dermis, fat and muscle) change with the variation of wavelength, while the variations of epidermis parameters are so small that can be ignored [21]. Additionally, the dispersive effect of human skin was ignored in our simulation, and the refractive index was considered as a parameter which did not vary with wavelength [25,26].

## 4. Simulation Results and Discussion

In order to verify the feasibility of the proposed method, the propagation of incoherent light in multi-layered skin tissues has been simulated using the Monte Carlo (MC) method. Compared with the method based on diffusion theory, MC method has comparatively high accuracy [27], and is considered as an effective universal method for the simulation of light propagation in turbid media [28,29].

### 4.1. Simulation Conditions

#### 4.1.1. Safety

Safety is obviously very important for any recharging method for implantable biosensors. In the proposed method, all the parameters of the LED light source must meet the safety requirements of the human body. According to the report of World Health Organization (WHO) [30], the limitation of light radiation intensity on the human skin is related to the wavelength and radiation exposure time. Furthermore, according to the regulations issued by International Commission on Non-Ionizing Radiation Protection (ICNIRP), the radiation intensity on human skin corresponding to visible light and near infrared light (400–1400 nm) should be less than 2.0 × 10^3^ C_A_W/m^2^ (C_A_ is a factor referring to the light wavelength), while the radiation exposure time should be limited to 8.33 h [31].

#### 4.1.2. Light Source

In the simulations for verifying the structure design of the LED light source, we assume that the spectrum range of the LED with a power of 10 mW is 800–900 nm, and the central wavelength (*λ*_c_) is 850 nm. Meanwhile, five wavelengths were chosen, which include 800, 825, 850, 875 and 900 nm, and the proportions of the light with the five wavelengths were determined according to the power distributions of the LED. In our simulation, the output light of the LED with the wavelength of λ was considered as Gaussian beam, while the proportion of the light corresponding to λ is expressed as:

(3) $$f(\lambda )=a_{1}\cdot e^{-{\left(\frac{\lambda -b_{1}}{c_{1}}\right)}^{2}}$$

where *a*_1_ = 0.5494, *b*_1_ = 850 and *c*_1_ = 24.93. According to the above spectrum distribution, the proportions of the lights corresponding to the five wavelengths are 0.0098 (800 nm), 0.2010 (825 nm), 0.5494 (850 nm), 0.2010 (875 nm) and 0.0098 (900 nm), respectively. The ray tracing results showed that 82% of the power at the skin surface is higher than 8.2 mW. Moreover, the power distribution is comparatively uniform, while approximately 90% of the power as well as the comparatively high power (≥7.4 mW) locates in the central area with a radius of 10 mm, which are treated as the effective area in our simulation.

### 4.2. Influence of Optical Wavelength

The influences of optical wavelength on the propagation characteristics of the incoherent light in the human body are simulated, represented as the parameters of absorbed fraction, diffuse reflectance and specular reflectance. In our simulation, the incident power of skin tissue was set as 5 mW, while the radius of spot radius was set as 10 mm.

#### 4.2.1. The Overall Results

**Figure 5.**
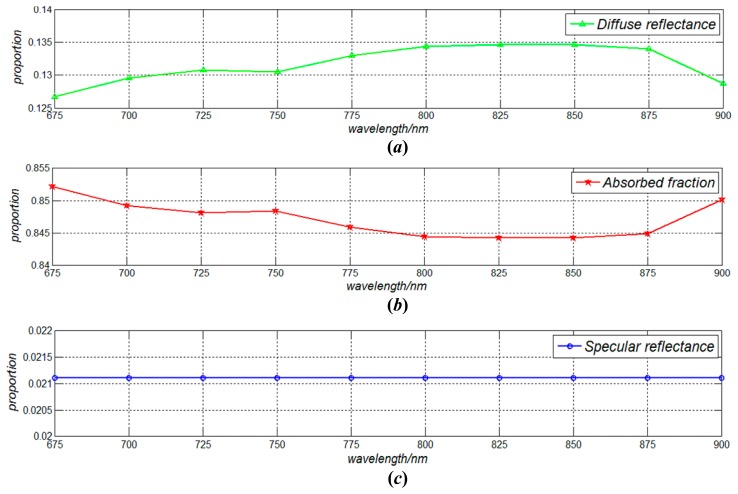
The influences of optical wavelength (800 nm–900 nm) to the (**a**) diffuse reflectance; (**b**) absorbed fractionand (**c**) specular reflectance in the light propagation within the human body.

Figure 5 shows our simulation results with respect to the influences of optical wavelength (800–900 nm) to the diffuse reflectance, absorbed fraction and specular reflectance in the light propagation within skin tissue.

#### 4.2.2. The Results of Each Layer

**Figure 6.**
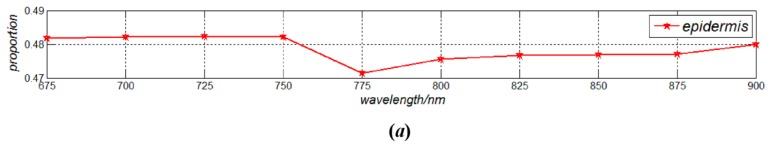

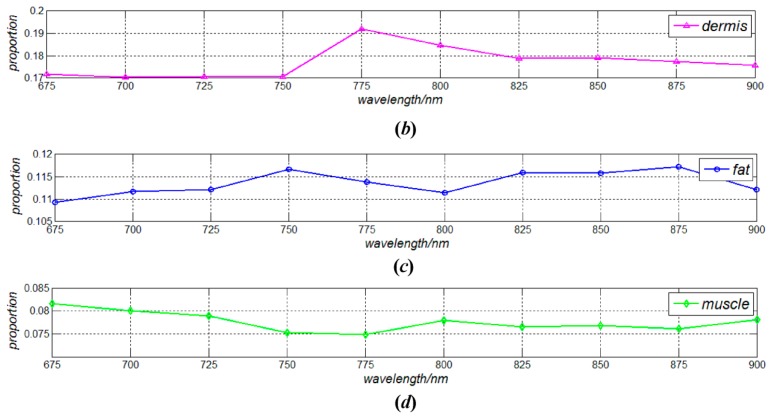
The absorbed fraction of tissue layers (epidermis, dermis, fat and muscle) corresponding to the different optical wavelengths (675–900 nm) in the propagation of incoherent light within the human body.

Figure 6 shows the absorbed fraction of each layer (epidermis, dermis, fat and muscle) corresponding to the different optical wavelengths in the propagation of incoherent light within the human body. As shown in Figure 6, the absorbed fractions of the epidermis, dermis, fat and muscle layers vary to some extent in the optical wavelength range of 675–900 nm. More concretely, the absorbed fraction of the epidermis layer is within the range of 0.471 (675 nm)–0.482 (850 nm). Moreover, the absorbed fraction of the dermis layer is within the range of 0.168 (900 nm)–0.192 (675 nm). On the other hand, the absorbed fractions of the fat and muscle layers are within the ranges of 0.109–0.145 and 0.063–0.082, respectively, which indicates that 6.3%–14.5% of the incident optical energy is absorbed in these two layers.

### 4.3. Distribution of the Energy

#### 4.3.1. Qualitative Analysis

The energy distributions within the human body of the incoherent light were achieved using weighted averaging method according to the spectrum distribution of the LED light source. More concretely, the energy distributions within the human body are expressed as the weighted average energy values corresponding to the wavelengths of 800, 825, 850, 875 and 900 nm.

**Figure 7.**
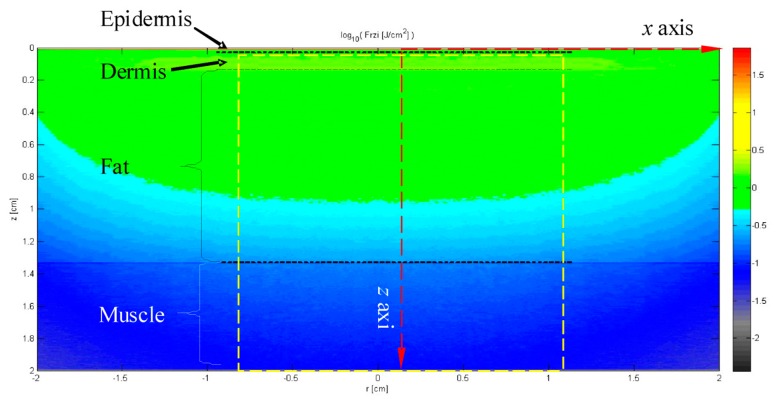
The distribution of energy density corresponding to the different depths of the human body.

Figure 7 shows the pseudo color chart with respect to the distribution of energy density, which is the simulation result using the developed model as well as the Monte Carlo method. As shown in Figure 7, the energy density has a symmetric distribution along the *z* axis, which corresponds to the spot center. On the other hand, the energy density has a comparatively stable distribution in the epidermis layer (*z* = 0.065−1.25 mm). Meanwhile, the energy density has an approximately elliptical distribution in the fat layer. In the area within the spot radius of 10 mm, it decreases almost linearly along the *z* axis. In the other area of the fat layer, the energy density decreases from the two focuses of the ellipse to the edge. Additionally, in the interface between the muscle layer and the fat layer, a clear dividing line can be found, while the energy density decreases gradually along *z* axis in the muscle layer (*z* = 13.31−2 mm).

#### 4.3.2. Energy Distribution along *x* Axis

**Figure 8.**
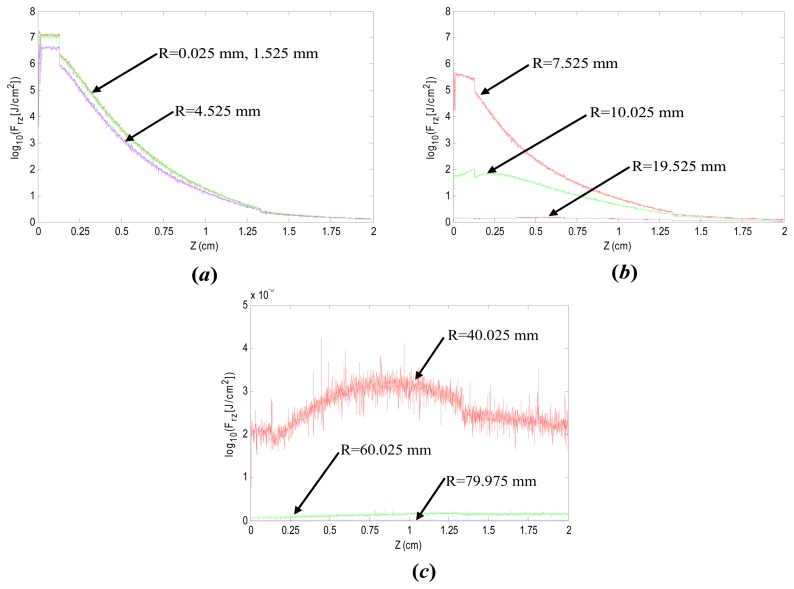
The distributions of energy density along *x* axis of tissue model.

In order to further reveal the distribution of the energy density of incoherent light in human tissue, the energy density of the achieved results along the *x* axis was also analyzed. Figure 8 shows the distributions of energy density corresponding to the nine spot radius (*R*) as well as the depths (*Z*) of tissue model, of which (a) *R* = 0.025 mm, 1.525 mm, 4.525 mm, (b) *R* = 7.525 mm, 10.025 mm, 19.525 mm, and (c) *R* = 40.025 mm, 60.025 mm, 79.975 mm, respectively, while *Z* is within the range of 0–20 mm.

As shown in Figure 8a, in the case that *R* < 4.525 mm, all the energy densities of the coherent light decrease gradually from 7.3 × 10^−2^ J/mm^2^ to 1.2 × 10^−3^ J/mm^2^ as the depths (*Z*) increase from 0 to 20 mm, which indicates that *R* has little effect on the energy density in this case. As shown in Figure 8b, in the case that 7.525 mm < *R* < 19.525 mm, the energy density corresponding to *Z* = 0 decreases from 7.0 × 10^−2^ J/mm^2^ to 3.5 × 10^−4^ J/mm^2^ as *R* increases from 7.525 to 19.525 mm. Meanwhile, all the energy densities decrease gradually with the increasing value of *Z*. Finally, all the values corresponding to *Z* = 20 mm are approximately 1.0 × 10^−5^ J/mm^2^. As shown in Figure 8c, in the case that 40.025 mm < *R* < 79.975 mm, the energy densities are very small, while the average values corresponding to three curves are 2.6 × 10^−5^ J, 1.3 × 10^−6^ and 6.0 × 10^−8^ J/mm^2^, respectively, which can be ignored. According to the above results, in order to achieve more energy for implantable biosensors, the radius of the photosensitive surface of the photovoltaic cell should be less than the spot radius (*d*/2) on the epidermis surface in the proposed method, and it is better to set the radius of the photosensitive surface of the photovoltaic cell to *d*/4.

#### 4.3.3. Energy Distribution along *z* Axis

**Figure 9.**
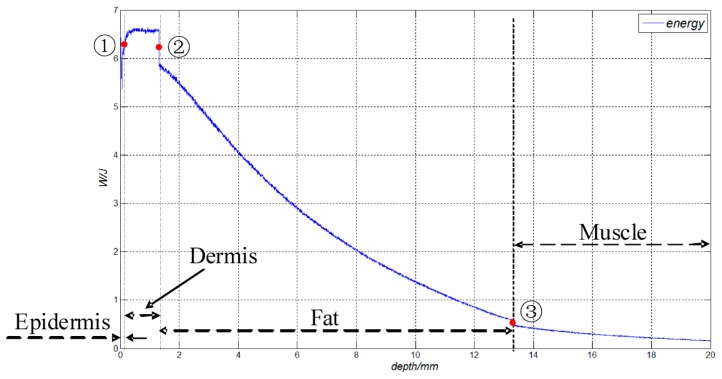
The distribution of the energy corresponding to the different tissue layers along *z* axis.

According to the achieved energy density results, the corresponding energy distribution can also be achieved by using an integral computing method. In our simulations, function interpolation was used to make the weighted average values of the energy density distributions more continuous, then the energy values were obtained by using the surface integral at different depths of the tissue layers. Figure 9 shows the power values obtained, whose incident optical energy is 10 J.

As shown in Figure 9, in the epidermis layer, the received energy increases from 6.617 to 5.371 J with increasing incident depth (*Z*), while point 1 (*z* = 0.065 mm) represents the interface between the epidermis layer and the dermis layer. In the dermis layer, the energy remains approximately stable and its average value is 6.534 J. Meanwhile, point 2 (*z* = 1.315 mm) represents the interface between the dermis layer and the fat layer. Moreover, since the relative refractive index (*n_rel_*) of the dermis layer is bigger than that of the fat layer, the corresponding decreasing of energy can be found at point 2. In the fat layer, the energy decreases from 5.833 to 0.591 J with the increasing of *z* from 1.25 to 12 mm. Similarly, the corresponding decreasing of energy can be found from the interface (point 3) between the fat layer and muscle layer. Finally, as *z* increases from 12 to 20 mm, the energy of the muscle layer decreases from 0.484 to 0.160 J, which indicates that there is approximately 0.5 J optical energy arriving at the muscle layer.

## 5. Experiment Results and Discussion

**Figure 10.**
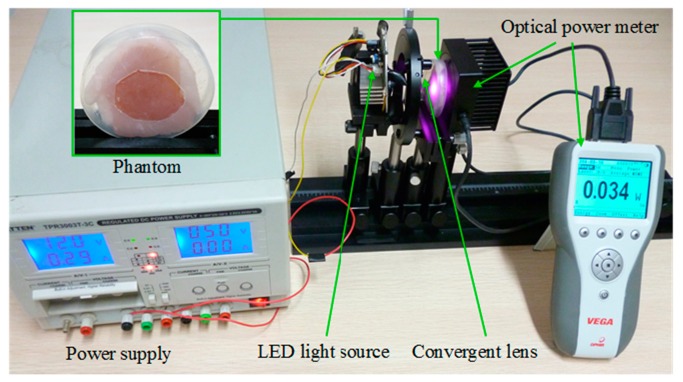
Experiment device of the recharging method based on LED light source.

In order to verify the validity of the proposed method, an experiment of the recharging method based on LED light source was carried out. The experimental device is composed of a LED light source (*λ*_c_ = 850 nm), a convergent lens (*f* = 25 mm, *F* = 1), a phantom (a piece of pork with the thickness of 6 mm), an optical power meter (active diameter 17.5 mm, spectral range 0.19–20 μm) and a power supply, as shown in Figure 10.

In our experiment, the optical power at the surface of the phantom is 332 mW. Given that the active diameter of the sensor of the optical power meter is 17.5 mm, the power density is 1.38 mW/mm^2^, which meets the safety regulation of ICNIRP (2.0 × 10^3^ W/m^2^). The power at the back of the phantom has been measured. As shown in Figure 10, the optical power meter shows that the power at the back of the phantom is 34 mW. Given that the efficiencies of photovoltaic cells generally are within the range of 10%–20% [32], 3.4–6.8 mW electric power can be achieved using the proposed method in the experiment. According to the above results, the advantages of the recharging method based on LED light source can be summarized as in Table 1, which shows the comparison between the proposed method and the other methods [4,6,8,10,12–15].

## 6. Conclusions

A recharging method for implantable biosensors based on a LED light source has been simulated and verified experimentally in this paper. We firstly develop models of the body tissue and the wearable LED light source. Secondly, the recharging processes of the proposed method have been simulated and tested, and the results have been analyzed and discussed. Some conclusions can be reached, as follows:

(1) In the proposed method, the power density should be less than 2.0 × 10^3^ C_A_W/m^2^. This safe condition can be achieved by setting the powers of light source and spot diameters on the skin surface.
(2) Both the diffuse reflectance and the absorbed fraction of the incoherent light increase with the increasing of optical wavelength from 675 to 850 nm, and decrease with the increasing of optical wavelength from 850 to 900 nm. However, the specular reflectance almost does not vary with the optical wavelengths.
(3) The absorbed fractions of the fat and muscle layers are within the ranges of 0.109–0.145 and 0.063–0.082, respectively, which indicates that 6.3%–14.5% of the incident optical energy can arrive at these two layers under the simulation conditions.
(4) The energy density has an approximately elliptical distribution in the fat layer. It decreases almost linearly along the *z* axis in the area within the spot radius of 10 mm. Meanwhile, the energy density decreases gradually along the *z* axis in the muscle layer (*z* = 13.31−2 mm).
(5) In order to provide more energy for implantable biosensors, the radius of the photosensitive surface of the photovoltaic cell should be less than the spot radius (*d*/2) on the epidermis surface, and it is better for setting the radius of the photosensitive surface of the photovoltaic cell to *d*/4.
(6) In the fat layer, the energy decreases from 5.833 to 0.591 J as *z* increases from 1.25 to 12 mm. Meanwhile, when *z* increases from 12 to 20 mm, the energy of the muscle layer decreases from 0.484 to 0.160 J, which indicates that approximate 0.5 J of optical energy can arrive at the muscle layer under the mentioned conditions.

Finally, according to the above discussions, we believe that the proposed method will help provide low-cost, convenient and safe method for recharging implantable biosensors, which will help to promote the applications of this kind of device in the future.

## Figures and Tables

**Table 1.** Comparison between the discussed method and the other methods.

**Methods**	**Safety**	**Returned Power (mW)**	**Size (cm^2^)**	**Convenience**	**Cost**	**References**
Radio frequency electromagnetic induction method	Low	10	Unknown	Yes	Low	[14]
Recharging method based on nanowires	Low	0.05	Unknown	No	Unknown	[4,6]
Thermo electric generator method	Low	0.001	1.0	Unknown	High	[8,10–12]
Previous optical recharging method	Low	4.00	10	No	High	[8,15]
The proposed optical recharging method	High	3.4∼6.8 mW	Unknown	Yes	Low	

**Table 2.** The parameters corresponding to the different wavelengths of the multi-layer tissues.

	**λ (nm)**	***μ****_a_* **(cm**^−^**^1^)**	***μ****_s_* **(cm**^−^**^1^)**	**g**	***n****_rel_* **(cm^−1^)**

Epidermis (d = 0.065 mm)	800−900	55	450	0.8	1.34
Dermis (d = 1.25 mm)	900	0.134	16.30	0.89	1.55
	875	0.122	16.98	0.88	
	850	0.122	17.57	0.87	
	825	0.121	18.24	0.87	
	800	0.127	19.07	0.86	

Fat (d = 12 mm)	900	0.125	10.88	0.59	1.45
	875	0.091	10.97	0.6	
	850	0.086	11.09	0.62	
	825	0.085	11.12	0.63	
	800	0.083	11.09	0.64	

Muscle (d = 1.0 × 10^8^ mm)	900	0.393	6.32	0.85	1.37
	875	0.368	6.43	0.85	
	850	0.343	6.60	0.85	
	825	0.309	6.78	0.85	
	800	0.284	6.60	0.85	
